# Curative Liver and Kidney Transplantation for Ongoing Symptoms of Acute Intermittent Porphyria and End‐Stage Kidney Disease

**DOI:** 10.1155/crit/2931133

**Published:** 2026-11-08

**Authors:** Siddarth Dasari, Deepika Suresh, Alden Doyle, Curtis Argo, Karl E. Anderson, Joseph Leeds

**Affiliations:** ^1^ University of Virginia School of Medicine (UVA) Charlottesville Virginia USA; ^2^ Division of Gastroenterology and Hepatology University of Virginia (UVA) Charlottesville Virginia USA; ^3^ Division of Nephrology University of Virginia (UVA) Charlottesville Virginia USA; ^4^ Division of Gastroenterology and Hepatology University of Texas Medical Branch Galveston Texas USA utmb.edu

## Abstract

Acute intermittent porphyria (AIP), the most common of the acute hepatic porphyrias, is a rare autosomal dominant disorder with variable penetrance resulting from pathogenic variants of the third enzyme in the heme biosynthetic pathway. Most heterozygotes never develop symptoms; some have infrequent acute attacks of multiple neuropathic manifestations including abdominal pain and motor weakness, and a few experience repeated debilitating attacks over many years. Long‐term complications may include end stage renal disease (ESRD), treated by dialysis or kidney transplantation, and liver cancer. Acute attacks are treated with intravenous hemin and may be prevented by prophylactic hemin infusions or givosiran, a long‐acting interfering RNA therapeutic. Liver transplantation is curative when medical therapies are inadequate. Simultaneous liver‐kidney transplantation (SLKT) has been reported to be beneficial in patients with ongoing active AIP and chronic kidney disease, but experience is limited. We report the case of a woman with AIP manifested by frequent recurrent attacks and chronic symptoms starting at age 17 and progressive renal disease beginning at age 28. Attacks were partially prevented by prophylactic hemin from age 29–42 and then givosiran from age 42–47. SLKT at age 47 restored renal function and resolved all biochemical and clinical manifestations of AIP. This experience supports SLKT as a suitable option for patients with ESRD and ongoing manifestations of AIP that respond incompletely to pharmacologic therapies.

## 1. Introduction

Acute Intermittent Porphyria (AIP) is a rare dominantly inherited disorder of heme biosynthesis due to the partial deficiency of porphobilinogen deaminase (PBGD), also known as hydroxymethylbilane synthase (HMBS) [1, 2]. This enzyme deficiency results from heterozygous pathogenic *HMBS* variants, of which > 300 have been identified in different families. Although most heterozygotes remain asymptomatic, attacks of neurological symptoms occur mostly in adult women because of induction of hepatic *δ*‐aminolevulinic acid (ALA) synthase 1 (ALAS1), the initial and rate‐limiting enzyme of this pathway in the liver. Certain drugs, hormonal steroids, and nutritional alterations are known to induce hepatic ALAS1, which leads to accumulation of pathway intermediates, including ALA and porphobilinogen (PBG), which are potentially neurotoxic. In some patients, attacks occur frequently even with avoidance of known inducing factors. Attack manifestations may include neuropathic abdominal pain, nausea, vomiting, tachycardia, hypertension and other autonomic features, hyponatremia, and neuropsychiatric symptoms. Chronic symptoms may persist after repeated attacks, and long‐term complications may include liver cancer (hepatocellular‐ or cholangiocarcinoma) and progressive kidney injury leading to ESRD [3, 4].

Acute attacks are treated by identifying and removing inducing factors such as drugs that induce hepatic ALAS1, carbohydrate loading and hemin infusions, which repress synthesis of hepatic ALAS1 and symptom management. Efforts to prevent attacks may include prophylactic hemin infusions [5], GnRH analogues for cyclic attacks [6, 7], and more recently, givosiran, a long‐acting siRNA therapeutic that targets ALA synthase 1 mRNA, thereby reducing neurotoxic precursor production [8]. Liver transplantation eliminates the source of overproduction of porphyrin precursors in this hepatic porphyria and offers metabolic cure in cases refractory to medical interventions [9].

Mechanisms of chronic kidney injury in AIP are incompletely understood and may be multifactorial. Elevated serum biomarkers for proximal tubular damage and inflammation have been reported in AIP [10, 11]. Persistent hypertension in AIP patients may result from catecholamine elevations and contribute to long‐term kidney damage [12]. Vasoconstriction during acute attacks may damage renal arterioles and lead to glomerular and interstitial damage [13]. Tubulointerstitial damage may also result from proximal tubular reabsorption of ALA by human peptide transporter 2 (PEPT2) [14]. Excessive amounts of infused hemin can cause acute kidney damage [15]. Monitoring of renal function is recommended during treatment with givosiran, although early small increases in creatinine have been reversible or stable with continued treatment [16].

Development of ESRD can worsen neuropathy and other manifestations of AIP and can be successfully managed by long‐term dialysis or renal transplantation. Renal transplantation is considered the treatment of choice and can also decrease attack frequency, perhaps by enhancing renal excretion of porphyrin precursors [17]. However, many AIP patients who underwent renal transplantation did not have high attack rates before transplantation. For example, in a series of 12 patients with AIP, one with latent AIP continued to have no attacks after renal transplantation and while attack rates decreased in the remaining 11, only one had an annualized attack rate greater than four prior to transplantation [18]. Therefore, SLKT may not be needed in most AHP patients considered for renal transplantation. However, the present case and four others previously reported indicate that SLKT may be beneficial in patients with ESRD and AIP with a high‐annualized attack rate that continues to respond incompletely to medical therapy [19–21].

## 2. Case Report

The patient was referred to the transplant clinic at the University of Virginia Medical Center, Charlottesville, Virginia at age 47 with CKD that had progressed to an eGFR of 13–14 mL/min and uremic symptoms including fatigue and a need for chronic peritoneal dialysis. She was diagnosed with AIP in Ukraine at the time of her first attack at age 17. A recurrent attack at age 18 was manifested by severe abdominal pain, seizures, and paresis progressing to quadriplegia with only partial movement of the right arm. After treatment with hemin (available in Europe as heme arginate [Normosang, Recordati]), she recovered over the next 3 months. She moved to Colorado at age 21 where attacks recurred every 4–5 weeks requiring multiple hospitalizations. Attacks were treated with glucose and later hemin (available in the United States as hematin [Panhematin, Recordati]). An intravenous port was inserted at age 22 for hemin treatment and subsequently required replacement many times. AIP was documented by elevations of urinary ALA [23.7 mg/g creatinine (ref < 7)], PBG [43.3 mg/g creatinine, (ref < 4)], and porphyrins 897 nmoL/g creatinine (ref < 300), reduced erythrocyte PBG deaminase (PBGD) activity [13 nmoL/mL/h (ref 20–50)], and a pathogenic insertion variant of the *PBGD/HMBS* gene (C.863‐864insT). She was treated with a GnRH analog (Lupron, Abbvie) with low‐dose estrogen add‐back for 6 years with little benefit. At age 27, she underwent hysterectomy and bilateral salpingo‐oophorectomy for stage 3B ovarian carcinoma followed by six cycles of carboplatin and paclitaxel and 12 cycles of maintenance paclitaxel which completed at age 30 with complete remission. Prophylactic hemin every 7 days was started at age 29 and successfully prevented major attacks for many years. As a nurse she was able to self‐administer hemin at home. She sometimes delayed hemin dosing until the onset of first attack symptoms, which then responded rapidly. Therefore, this hemin regimen was in part on demand rather than prophylactic. Minor attacks were managed at home ~10 times per year and major attacks requiring hospitalization several years apart. Milder chronic symptoms between attacks especially after about age 35 included abdominal pain and numbness and tingling in fingers and toes, which were managed symptomatically, including low‐dose opioids. This treatment continued when she moved to Florida, Texas and finally to Virginia at age 44. High ferritin levels noted since age 30 might have been due to an ongoing acute phase response to frequent doses of hemin, but suggested iron overload and prompted treatment with deferasirox for several years. Obesity related at least in part to efforts to maintain a high glucose oral intake for AIP progressed to 283 lbs and was associated with obstructive sleep apnea treated with CPAP since age 24. Other conditions included hypertension, prediabetes, gout affecting the left foot, chronic, mild transaminase elevations and hepatic steatosis on liver imaging first noted at age 38, hypothyroidism, depression, and repair of ventral hernia at age 37. Persistent transaminase elevations prevented consideration for initial clinical trials with givosiran, but after its FDA approval monthly givosiran (Givlaari, Alnylam) injections were started at age 42, after which attack frequency decreased and an on demand hemin dose was needed only at intervals of several months.

Increased serum creatinine was first noted at age 28 and progressed gradually, which was attributed to porphyria‐associated nephropathy, hypertension, and obesity. At age 35, her serum creatinine was 1.0–1.2 mg/dL with eGFR 46.9 ≥ 50 mL/min with trace protein intermittently on urinalysis. At age 42, creatinine was 1.8 mg/dL with eGFR of 30.7 mL/min with proteinuria of 1.066 mg/g creatinine. Her creatinine continued to increase after starting givosiran, from 1.9 mg/dL at age 43 to 2.5 mg/dL with eGFR of 25.4 mL/min and proteinuria of 1.47 g/g creatinine ratio at age 44. Although givosiran can cause a decrease in kidney function, this is described as usually transient and nonprogressive and difficult to distinguish from other causes of decline in renal function [8, 16, 22]. Kidney biopsy at age 43 and shortly after starting givosiran showed adaptive focal segmental glomerular sclerosis with glomerulomegaly and preserved podocyte foot processes, arterio‐ and arteriolar nephrosclerosis with hyaline sclerosis with 47% global glomerulosclerosis and 40% interstitial fibrosis and tubular atrophy, which was highly consistent with advanced porphyria‐associated kidney disease [3, 13] and considered to explain her progressive decline in renal function. At age 43, hypertension and proteinuria were managed with hydrochlorothiazide, labetalol, dapagliflozin, and losartan. At age 46, she was referred for transplant evaluation with Stage V CKD currently maintained on peritoneal dialysis for 1 year prior to transplant. Full timeline in Figure 1.

**Figure 1:**
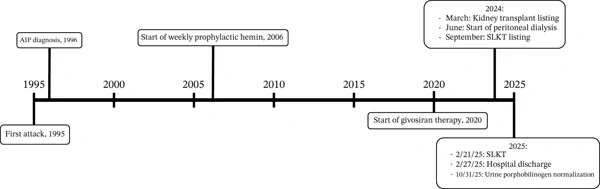
Timeline of the patient′s clinical course and interventions for refractory AIP. Events include first attack, diagnosis confirmation, medical therapies, transplant listings, dialysis initiation, SLKT, and posttransplant events.

It was decided to pursue SLKT and AIP cure rather than KT alone in this patient because of her long history of severe recurrent AIP symptoms that were incompletely controlled by prolonged preventive treatment with hemin followed by givosiran and multiple associated medical problems. The aim was to eliminate future attacks and other consequences of AIP and any need for future treatments with hemin or givosiran. Both treatments had been beneficial but incompletely effective for management of her AIP symptoms. There was also concern that with KT alone continued treatment with givosiran would be needed to reduce attack frequency, and this might contribute to future damage to the kidney graft. Moreover, AIP cure would likely enable more effective management of her other medical problems such as hypertension and obesity. At pretransplant evaluation, risk factors for metabolic associated steatotic liver disease (MASLD) included morbid obesity with a history of gastric sleeve surgery, hypertension, hyperlipidemia, and elevated glucose without overt diabetes but with normal liver function tests at that time and borderline thrombocytopenia. Her MELD 3.0 score was 21, largely driven by renal dysfunction, Fib‐4 score was intermediate (1.68), and liver biopsy was not pursued after screening tests for other causes of chronic liver diseases were negative.

Six months after listing, at age 47, she underwent successful SLKT from a brain death (DBD) donor with KDPI 7%. The peritoneal dialysis catheter was removed and the liver and kidney grafts were implanted sequentially without intraoperative complications. Intraoperative fluid administration included 4 L of crystalloid and 2 L of colloid. The patient received 2 units of packed red blood cells (pRBCs), and cell salvage yielded 240 mL. Estimated blood loss was 1 L, and intraoperative urine output was 670 mL. A peritoneal dialysis fluid sample at surgery grew Bacillus species, which was treated with a 7‐day course of piperacillin–tazobactam transitioned to amoxicillin–clavulanate with no adverse consequences. A fluid bolus was required on postoperative Day (POD) 1 for decreased urine output. Induction immunosuppression was initiated with intraoperative methylprednisolone and basiliximab, and tacrolimus and mycophenolate mofetil were started on POD 0. The explanted liver showed mild macrovesicular steatosis (5%–10%) and early periportal fibrosis. The liver graft functioned immediately, and liver enzymes remained normal. The kidney allograft was considered to have slow graft function but dialysis was weaned and creatinine steadily improved and she was discharged off dialysis on POD 6. Discharge immunosuppression was tacrolimus for a goal of 8–10 ng/mL, mycophenolate mofetil 1000 mg BID, and prednisone taper to 5 mg daily. Ongoing preventive treatment included trimethoprim–sulfamethoxazole for pneumocystis jirovecii pneumonia and valganciclovir for cytomegalovirus prophylaxis as both donor and recipient were CMV seropositive. Givosiran treatment was not resumed after transplantation. One day after discharge, she was readmitted with new urinary incontinence and bladder spasms and after no structural abnormalities or infections were identified, these resolved with oxybutynin, methocarbamol, and heating pad outpatient therapy.

In the following weeks, she remained dialysis‐independent with stable creatinine (0.9–1.1 mg/dL), normal liver function, and no signs of rejection. Surgical drains were removed, and immunosuppressive drug levels remained within target ranges. Importantly, her urine PBG downtrended and has remained within normal limits 1.0 *μ*moL/24 h (Figure 2), and she experienced complete cessation of porphyric attacks for the first time in 20 years.

**Figure 2:**
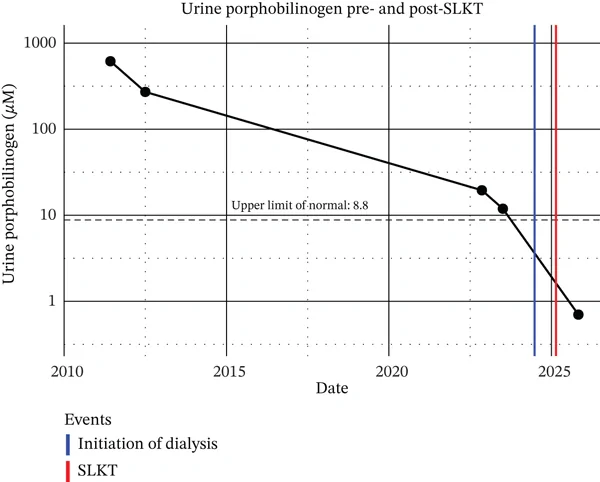
Longitudinal urine PBG levels in the patient across the clinical timeline. Measurements prior to transplant show markedly elevated PBG consistent with ongoing porphyric activity. The vertical blue line indicates initiation of peritoneal dialysis, whereas the vertical red line marks the date of SLKT. Following SLKT, PBG levels normalized, demonstrating resolution of metabolic dysfunction and cessation of porphyric attacks.

## 3. Discussion

This case and five previous reports, four described in detail (Table 1), demonstrate that SLKT can provide dialysis independence as well as durable metabolic cure for patients with ESRD and continuingly active AIP [9, 19–21]. Our patient had experienced frequent porphyric attacks beginning at age 17, which thereafter required long‐term prophylactic treatment with hemin or givosiran to achieve partial control of her symptoms. Progressive renal disease developed, which was attributable to AIP with contributions from hypertension and possibly givosiran. SLKT at age 47 has restored renal function and eliminated AIP symptoms completely and the need for partially effective preventive treatment with hemin or givosiran. Liver transplantation has also eliminated other risks, such as potential damage to the renal graft from active AIP and givosiran and risk for developing liver cancer. Cure of AIP is also allowing her to more effectively address her other medical problems, including obesity, other features of the metabolic syndrome, and reduce risk of developing steatosis in the liver graft.

**Table 1:** Features of four previously published cases and the present case of AIP who underwent SLKT, including demographics, specific *HMBS* variants, pretransplant clinical features and preventive treatments (prophylactic hemin or givosiran), and posttransplant responses. A fifth case was reported as successful but not described in detail [9].

Case	Authors/year	Age at transplant, sex	*HMBS* variant (type)	Pretransplant features	Preventive treatments	Posttransplant response
1.	Wahlin et al., 2010 [19]	24 F	104‐105insGTCT (insertion)	•Abdominal pain, hypertensive crises, episodes of motor neuropathy, and repeated hospitalization for many years	•Available preventive treatments (not specified) of little help	•At 16 months: no clinical or biochemical signs of AIP.
						•Slight residual weakness.
2.	Wahlin et al., 2010 [19]	55 F	593G>A (W198X) (missense)	•Worsening episodes of abdominal pain, motor weakness, and other symptoms for 10 years	•Not noted	•At 11 months: no clinical or biochemical signs of AIP
						•No evidence of neuropathy.
3.	de Sousa Arantes Ferreira et al., 2020 [20]	19 M	IVS9–1G>A (5 ^′^ donor splice site)	•Attacks of abdominal pain age 15–18	•None	•Improvement in motor weakness
				•Progressive CKD age 17, started on hemodialysis		
						•No further attacks
				•Severe motor weakness age 18		
4.	Petrides et al., 2025 [21]	20 F	c.445C>T, p.Arg199X (missense)	•Progressively more frequent attacks starting age 16, abdominal pain, motor neuropathy	•Regular heme infusions every 3 months, then monthly	•No clinical or biochemical signs of AIP
						•Preeclampsia, healthy premature delivery by C section, age 38
				•Iron overload, hepatic siderosis without fibrosis		
				•Opioid tolerance		
				•Port complications		
				•Parenteral nutrition for low body weight		•Iron deficiency
				•Hyperthyroidism		
				•Normal pregnancy age 22		
5.	This case	47F	c.863_864InsT (Insertion)	•Severe, frequent attacks since age 17 requiring preventive treatments	•Partial benefits from hemin prophylaxis and givosiran	•At 18 months: no clinical or biochemical signs of AIP

The first report of SLKT in AIP was from Sweden [19] and included two female patients with dialysis‐dependent ESRD and refractory manifestations of AIP including progressive neuropathy who underwent combined transplantation with complete resolution of clinical symptoms and normalization of porphyrin precursor levels following surgery. At that time givosiran was not available, but other preventive treatments were not helpful in one patient. The third patient was a 19‐year‐old male in Brazil with attacks from age 15–18 and severe motor weakness. Preventive treatment with hemin or givosiran was not available. After SLKT he had no further attacks and motor weakness improved [20]. The fourth case was a 20‐year‐old female in Germany with progressively more frequent attacks starting at age 16. She developed motor neuropathy, opioid tolerance, and iron overload because of numerous hemin infusions, but after SLKT she had no clinical or biochemical manifestations of AIP and had a successful pregnancy [21]. A fifth case was described as successful in a review of liver transplantation results in Europe but without further details [9]. Givosiran was not available for these prior cases.

Givosiran, an siRNA therapeutic that suppresses hepatic ALAS1 expression and reduces production of potentially neurotoxic porphyrin precursors, has transformed the management of AIP for many patients, but as in this patient may not achieve complete prevention of symptoms or delay long term complications such as renal disease [4, 18]. Although liver transplant alone is curative and can prevent further attacks, persistent CKD requiring chronic dialysis may complicate recovery and worsen long term outcomes of AIP [23]. Therefore, SLKT may be optimal for some AHP patients with both CKD and ongoing manifestations of porphyria.

Long‐term renal damage in AHP is thought to result from toxic effects of heme precursors, particularly ALA, chronic hypertension and from renal vasoconstriction and arteriolar damage during recurrent attacks. Renal biopsy in our patient demonstrated adaptive focal segmental glomerulosclerosis (FSGS), arteriolar nephrosclerosis with hyaline sclerosis, and 40% interstitial fibrosis and tubular atrophy, which was consistent with previous reports of advanced porphyria‐associated nephropathy [3, 13]. The liver explant in this case showed mild macrovesicular steatosis (affecting 5%–10% of hepatocytes) with early periportal fibrosis. These findings in the context of longstanding obesity and hyperlipemia suggest MASLD, which is common in the population and may have increased AIP activity long term in this patient. Hemosiderosis was not evident histologically in the explant liver, and elevated serum ferritin levels observed just before individual prophylactic hemin infusions may reflect an acute‐phase inflammatory response of unknown duration after preceding hemin infusions. Hepatic fibrosis was not evident on liver elastography, with only mild steatosis and periportal fibrosis seen on explant histopathology, so liver transplantation in this case was driven by AIP activity rather than structural liver disease.

## Funding

No funding was received for this manuscript.

## Consent

Written informed consent was obtained from the patient for publication of this case report and any accompanying images.

## Conflicts of Interest

The authors declare no conflicts of interest.

## Data Availability

The data that support the findings of this study are available on request from the corresponding author. The data are not publicly available due to privacy or ethical restrictions.
